# ABO Blood Type Is Associated with Thrombotic Risk in Patients with Nonvalvular Atrial Fibrillation

**DOI:** 10.3390/jcm11113064

**Published:** 2022-05-29

**Authors:** Albert Youngwoo Jang, Jeongduk Seo, Yae Min Park, Yong Hoon Shin, Joonpyo Lee, Pyung Chun Oh, Woong Chol Kang, Wook-Jin Chung, Jeonggeun Moon

**Affiliations:** 1 Division of Cardiology, Department of Internal Medicine, Gil Medical Center, Gachon University College of Medicine, Incheon 21565, Korea; cardio_gil@gilhospital.com (A.Y.J.); jaidyseo@gmail.com (J.S.); ypruimin@gilhospital.com (Y.M.P.); fibrillary@gilhospital.com (Y.H.S.); joonpyu@gilhospital.com (J.L.); likemed@gilhospital.com (P.C.O.); kangwch@gilhospital.com (W.C.K.); 2 Gachon Cardiovascular Research Institute, Gachon University, Incheon 21936, Korea

**Keywords:** ABO blood group, atrial fibrillation, risk factor, stroke, thromboembolism

## Abstract

Blood type is reportedly correlated with the occurrence of cardiovascular diseases, presumably because of its effect on thrombogenicity. However, the relationship between blood type and thrombotic complications in atrial fibrillation (AF) remains unclear. This retrospective study analyzed the blood types of 1170 AF patients (mean age, 70 years; 58% men) who were followed up for up to 4 years. Patients with greater than mild mitral stenosis or prosthetic valves were excluded. The cohort included 305 (26%) type O, 413 (35%) type A, 333 (28%) type B, and 119 (10%) type AB patients. The primary endpoint of major adverse cerebrovascular events (MACE) occurred in 52 (4.4%) patients. When longitudinal outcomes were plotted, AB blood type patients had worse prognosis than non-AB blood type patients (*p* = 0.039), particularly type O blood patients (*p* = 0.049). Multivariate Cox regression analysis revealed that AB blood type was associated with higher MACE rates (adjusted hazard ratio, 2.01; 95% confidence interval, 1.01–4.00; *p* = 0.048) than non-AB blood types independent of anticoagulation therapy duration or CHA2DS2-VASc score. These indicate that AF patients with AB blood type are at an increased risk of MACE compared to those with non-AB blood type independent of the duration of anticoagulation or the CHA_2_DS_2_-VASc score.

## 1. Introduction

Atrial fibrillation (AF) is the most common sustained cardiac rhythm disorder [1] and is associated with an increased risk of cardiac embolisms, including ischemic stroke [2] and cognitive dysfunction [3]. The CHA_2_DS_2_-VASc scoring system utilizes clinical features to predict thromboembolic events and guide anticoagulation therapy [4]. Despite such efforts, many additional mechanisms underlying thrombogenesis in AF have not been suggested, such as inflammation [5] or endothelial dysfunction [6], and the role of thrombogenesis remains unclear. Additionally, the risk of stroke remains high despite the most updated anticoagulation therapy. Thus, investigating factors associated with thrombotic tendency in AF is of crucial importance [7].

Blood type is reportedly related to cardiovascular diseases [8] and venous thromboembolism, with patients with type O blood being at a reduced risk [9]. However, to the best of our knowledge, the relationship between blood type and thrombotic tendency in AF has not yet been studied. This study aimed to identify the influence of blood type on thrombotic risk in patients with AF using real-world data.

## 2. Materials and Methods

### 2.1. Study Sample

**Figure 1:**
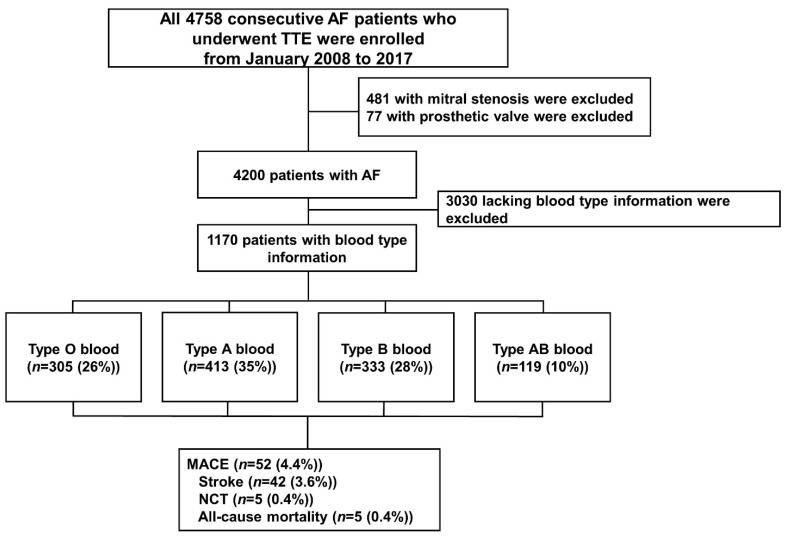
Diagram for enrollment. After the exclusion of 558 patients with valvular diseases, 4200 patients with nonvalvular atrial fibrillation were analyzed. A total of 1170 patients were enrolled after the exclusion of 3030 patients for whom no blood type information was available. AF, atrial fibrillation; NCT, non-cerebral thromboembolism; TTE, transthoracic echocardiography.

This retrospective observational study was approved by the Institutional Review Board of the Gil Medical Center, Gachon University College of Medicine (GDIRB2018-305) and conformed to the Declaration of Helsinki (6th revision). The medical records and transthoracic echocardiography database of patients with AF treated at the institution between 2011 and 2017 were analyzed. Korean patients with AF, aged ≥18 years, and with available blood type data (O, A, B, or AB) were included in the study. The exclusion criteria were mitral stenosis of greater than moderate severity on echocardiography or the presence of a prosthetic valve. Approximately 22% of patients were followed up for secondary prevention of systemic thromboembolism, including stroke, transient ischemic attack (TIA), or non-cerebral thromboembolism (NCT). Other patients visited our hospital for perioperative evaluations or regular health checkups. The patients’ demographic data have been described previously [5,10]. The detailed patient enrollment process is shown in Figure 1.

### 2.2. Clinical Outcome Assessment

The primary endpoint was major adverse cerebrovascular events (MACE), defined as a composite of ischemic stroke, NCT, and all-cause mortality. The secondary endpoints were ischemic stroke, NCT, and all-cause mortality.

### 2.3. Statistical Analyses

Data analysis was performed using IBM SPSS Statistics (version 23.0; IBM Corp., Armonk, NY, USA). Continuous normally distributed data are expressed as mean ± standard deviation, whereas continuous non-normally distributed data are presented as medians and interquartile ranges. Student’s one-way analysis of variance was used to assess inter-group differences in normally distributed variables, whereas Tukey’s post hoc test was used to determine inter-group statistical significance. Categorical variables were analyzed using the Pearson χ^2^ test and Mann–Whitney U test for normally and non-normally distributed data, respectively. We constructed a stepwise multivariable Cox proportional hazards regression model to evaluate the independent effect of blood type on the outcomes. Hazard ratios (HR) and 95% confidence intervals (CI) were also calculated. The CHA_2_DS_2_-VASc score was defined as an ordinal variable within each Cox regression multivariable model. Longitudinal data for the outcomes were plotted using the Kaplan–Meier estimates with the log-rank test. Statistical significance was set at *p* < 0.05.

## 3. Results

### 3.1. Baseline Characteristics

The baseline clinical characteristics are shown in Table 1. Type A blood was the most common (35%), whereas type AB was the least common (10%). The mean age of the patients was 70 years, 59% were men, and the mean CHA_2_DS_2_-VASc score was 2.79 ± 1.86. Congestive heart failure, hypertension, and diabetes mellitus were present in 19%, 17%, and 23% of the patients, respectively. At the time of study enrollment, approximately 46% of the patients were prescribed with anticoagulation therapy consisting of a vitamin K antagonist (26%) or novel oral anticoagulant (NOAC) (20%) at the attending physician’s discretion. A total of 468 (40%) patients had a CHA_2_DS_2_-VASc score of ≥2. Of note, the mean anticoagulation duration was 7.6 months during the mean 17-month follow-up period (Table 2). Furthermore, 22% of patients were followed for secondary prevention of thromboembolic events (TE), such as stroke, TIA, or other systemic/pulmonary TE. The prescription rate of oral anticoagulants (OAC) was consistent with previous Korean data during the corresponding period. The proportion of patients administered with vitamin K antagonists or NOAC, beta-blockers, angiotensin-converting enzyme inhibitors/angiotensin receptor blockers, or diuretics was comparable among all blood types.

### 3.2. Clinical Outcomes

**Figure 2:**
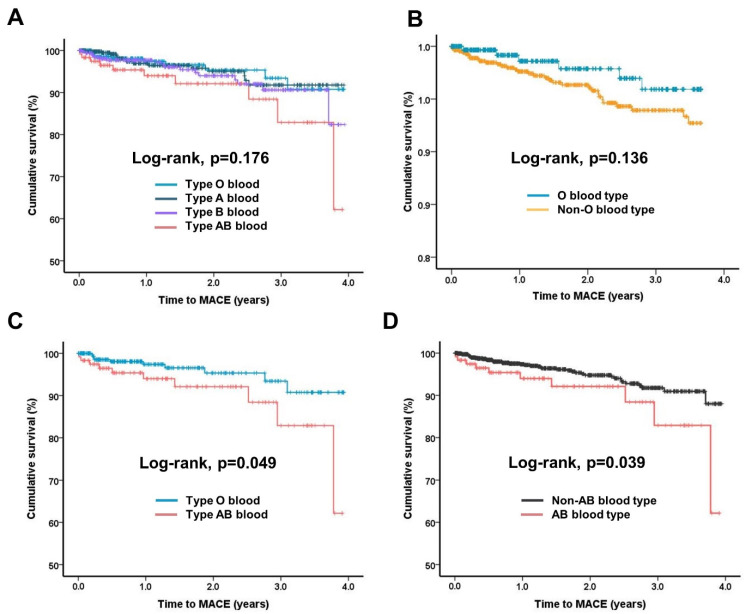
Kaplan–Meier survival analysis of different blood types. (**A**) All blood types; (**B**) O and non-O blood types; (**C**) O and AB blood types; (**D**) AB and non-AB blood types. MACE, major adverse cerebrovascular events.

Longitudinal clinical outcomes are presented in Table 2. Among the patients, 52 (4.4%) experienced MACE, of which 42 (3.6%), 5 (0.4%), and 5 (0.4%) experienced ischemic stroke, NCT, and death, respectively. The Kaplan–Meier survival plots for the different blood types are shown in Figure 2. There were no significant overall differences (*p* = 0.176) between the blood types (Figure 2A) although the AB group appeared to have worse outcomes in the subgroup analysis. As previous studies have claimed that individuals with a non-O blood type are at a decreased risk of cardiovascular disease [9], we first compared the prognosis of the O vs. non-O blood types. However, there were no significant differences (*p* = 0.139) although the non-O group had numerically better outcomes (Figure 2B). Our data suggested that patients with type AB blood had worse prognosis; thus, we performed further analysis comparing the outcomes of patients with AB and O blood types as well as AB and non-AB blood types. In the subgroup analysis, the AB blood type was associated with a significantly more frequent and earlier occurrence of MACE (*p* = 0.049) than the O blood type (Figure 2C); type AB blood was also predictive of higher rates and an earlier occurrence of MACE (*p* = 0.039) than the non-AB blood types (Figure 2D).

### 3.3. Multivariable Cox Regression for Analyzing Predictors for Long-Term Adverse Outcomes

Table 3 shows the multivariate Cox regression analysis of the predictors of MACE. AB blood type was an independent predictor (adjusted HR, 2.01; 95% CI, 1.01–4.00; *p* = 0.048) for MACE versus a non-AB blood type (Model 1 of Table 3). However, the presence of type O blood was not associated with MACE (adjusted HR, 0.75; 95% CI, 0.38–1.50; *p* = 0.422) compared with a non-O blood type (Model 2 of Table 3). In addition, the CHA_2_DS_2_-VASc scores were not associated with the occurrence of MACE in either model (*p* > 0.05). Of note, a longer duration of anticoagulation use was uniformly associated with an approximately 3% reduction in MACE for a 1-month increase in treatment duration in both models (adjusted HR, 0.97; 95% CI. 0.94–0.99; *p* = 0.008).

## 4. Discussion

This retrospective observational study based on real-world data demonstrated that type AB blood was associated with thrombotic events in patients with AF independent of CHA_2_DS_2_-VASc scores and antithrombotic treatment duration. In this study, the O blood type appeared to be associated with better event-free survival than the other blood types [9,11,12] although the difference was not statistically significant (Figure 2B). The O blood type also showed significantly better prognoses than the AB blood type (Figure 2C). Notably, the incidence of MACE was significantly higher in AB blood type patients than in non-AB blood type patients during follow-up. Although it is unclear why AB blood type patients showed a worse prognosis solely based on data from the current investigation, our findings are consistent with those of other studies regarding the influence of blood type on venous thromboembolism [13]. Currently, many questions regarding the role of blood type in the pathogenesis of cardiovascular diseases remain unanswered. However, it is clear that blood type is associated with thrombotic diseases, albeit without a clearly proven mechanism [14].

The CHA_2_DS_2_-VASc score, a strong predictor of stroke in patients with AF, failed to show an association with clinical outcomes in our cohort for several reasons. First, anticoagulation therapy was not well controlled in our study sample and could have served as a confounder. Approximately 46% of all patients were prescribed with OAC at the start of follow-up. The OAC prescription rate in patients with a CHA_2_DS_2_-VASc score of ≥2 was 48%, which was significantly lower than that in dedicated global registries [15,16] and guideline recommendations [17]. However, the underuse of OAC in our cohort is consistent with other real-world cohorts during the similar era [15,18,19]. The low prescription rate of NOAC (24% in CHA_2_DS_2_-VASc ≥ 2) was probably because most patients were enrolled in the pre-NOAC era; the use of NOACs was approved by the Korean Food and Drug Administration in 2015. Second, patient adherence to those medications was poor (only 7.6 months of the mean follow-up period of 17 months), which implies the attending physicians’ suboptimal alertness toward thromboprophylaxis or difficulty maintaining long-term anticoagulation therapy because of issues such as hemorrhagic complications. However, it is noteworthy that a longer anticoagulation therapy duration was significantly associated with reduced ominous outcomes in our study patients (Table 3). This indicates that drug adherence is important to prevent such outcomes in patients with AF. Currently, blood type is not a clinically recognized factor for prescribing anticoagulation therapy in real-world practice. As suggested by our data, blood type may be a predictor of thrombotic events independent of the CHA_2_DS_2_-VASc score and anticoagulation therapy duration. Further investigation in a validation cohort is required to confirm these findings.

ABO blood-type antigens (i.e., A, B, and H) are complex terminal glycan structures on glycolipids and glycoproteins formed by the addition of N-acetylgalactosamine (A antigen) or D-galactose (B antigen) onto a core H antigen via ABO glycosyltransferase action [20]. For decades, it has been known that patients with type O blood have a reduced risk of venous/arterial thrombotic events, including myocardial infarction, coronary artery disease, ischemic stroke, and peripheral vascular disease [21,22,23]. A suggested mechanism for the relationship between blood type and thrombosis is the von Willebrand factor (vWF) action [24,25]. The ABO blood-type antigens on vWF are located near the residues where a disintegrin and metalloproteinase with thrombospondin type 1 repeats-13 (ADAMTS13) cleave vWF. ADAMTS13 proteolytic efficiency is affected by neighboring ABO antigens, which results in lower proteolytic rates in individuals with a non-O blood type [26]. Hence, the plasma vWF level is 25% higher in individuals with a non-O blood type [25]. Similarly, an increased coagulation factor VIII level in individuals with a non-O blood type due to the same mechanism is also responsible for increased thrombogenesis [25]. The genetic linkage of the ABO locus with low-density lipoprotein, type 2 diabetes, and inflammatory risk biomarkers, such as E-selectin, P-selectin, and intercellular adhesion molecules, could at least partially explain the higher prevalence of cardiovascular disease and/or adverse lipid profiles in patients with a non-O blood type [27,28,29]. In addition, ABO antigens are expressed on the surfaces of a variety of cells and tissues, including red blood cells, platelets, sensory neurons, epithelial cells, and vascular endothelial cells, as well as on some plasma proteins such as vWF. Thus, ABO blood types are reportedly associated with venous thrombosis as well as various conditions such as cancer, preeclampsia, cognitive dysfunction, metabolic diseases, and infectious disorders [21,30,31,32,33,34,35]. From a different perspective, the ABO gene locus has been shown to influence angiotensin-converting enzyme activity [36,37]; this association suggests a potential relationship between blood type and the risk of heart failure. A recent study showed the prognostic significance of blood type in patients with heart failure [38].

This study has the inherent limitations of being a single-center retrospective observational study performed using chart reviews at a single center. We also acknowledge that the study was significantly undersized to derive strong conclusions although it may provide novel insights to future research. In addition, the patient population was biased, as all patients were referred to a tertiary hospital for various reasons. Approximately 15% of the patients were evaluated preoperatively, which led them to be followed up for a short period after surgery, thus leading to a follow-up period with a wide standard deviation. All patients were diagnosed and treated in a tertiary referral hospital; therefore, the study sample was not free of selection bias. Although all patients were enrolled in a single center, they were treated by multiple attending cardiologists; therefore, their treatments and anticoagulant prescriptions were not standardized. Even though the overall sample size was fairly large, low event rates, possibly due to follow-up loss or prescription from other hospitals, may be a limitation.

In conclusion, our findings demonstrate that blood type is associated with MACE in patients with AF, with the lowest risk associated with type O and the highest risk associated with type AB. This relationship is independent of the CHA_2_DS_2_-VASc score or the duration of anticoagulation therapy. These real-world data provide additional evidence of the association between blood type and thrombotic cardiovascular disease.

## Figures and Tables

**Table 1:** Baseline characteristics and outcomes of the total cohort.

	All (*n* = 1170)	Type O Blood (*n* = 305 (26%))	Type A Blood (*n* = 413 (35%))	Type B Blood (*n* = 333 (28%))	Type AB Blood (*n* = 119 (10%))	*p*
**Demographic data**						
Age (years)	70 ± 11	70 ± 11	70 ± 11	70 ± 11	72 ± 9	0.026
Men, *n* (%)	673 (58)	175 (57)	233 (56)	201 (60)	64 (54)	0.575
Previous medical history						
Congestive heart failure, *n* (%)	222 (19)	71 (23)	63 (15)	61 (18)	27 (23)	0.036
Hypertension, *n* (%)	197 (17)	51 (17)	74 (18)	49 (15)	23 (19)	0.584
Diabetes mellitus, *n* (%)	264 (23)	71 (23)	91 (22)	72 (22)	30 (25)	0.849
Secondary prevention for TE, *n* (%)	257 (22)	61 (20)	99 (24)	73 (22)	24 (20)	0.725
Ischemic stroke, *n* (%)	195 (17)	54 (18)	71 (17)	54 (16)	16 (13)	0.740
TIA, *n* (%)	25 (2)	9 (3)	5 (1)	9 (3)	2 (2)	0.349
Systemic/pulmonary TE, *n* (%)	40 (3)	7 (2)	14 (13)	15 (5)	4 (3)	0.502
Peripheral arterial disease, *n* (%)	26 (2)	8 (3)	8 (2)	8 (2)	2 (2)	0.899
Myocardial infarction, *n* (%)	54 (5)	12 (4)	18 (4)	19 (6)	5 (4)	0.722
CHA_2_DS_2_-VASc score	2.79 ± 1.86	2.82 ± 1.82	2.79 ± 1.83	2.72 ± 1.92	2.95 ± 1.87	0.712
**Concurrent medication**						
OAC, *n* (%)	541 (46)	139 (46)	192 (46)	153 (46)	57 (48)	0.860
VKA, *n* (%)	301 (26)	75 (25)	111 (27)	82 (25)	33 (28)	
NOAC, *n* (%)	240 (21)	64 (21)	81 (20)	71 (21)	24 (20)	
OAC in CHA_2_DS_2_-VASc ≥ 2, *n* (%)	403 (48)	99 (44)	136 (46)	109 (47)	42 (47)	0.737
VKA, *n* (%)	204 (24)	49 (22)	72 (26)	55 (24)	24 (27)	
NOAC, *n* (%)	199 (24)	52 (23)	64 (22)	54 (24)	18 (20)	
Duration of anticoagulation (months)	7.6 ± 13.7	7.2 ± 13.2	7.9 ± 14.4	8.0 ± 13.9	6.9 ± 12.4	0.788
Beta-blockers, *n* (%)	332 (28)	84 (28)	127 (31)	88 (26)	33 (28)	0.594
ACEi/ARBs, *n* (%)	458 (39)	120 (39)	167 (40)	131 (39)	40 (34)	0.609
Diuretics, *n* (%)	435 (37)	116 (38)	147 (36)	131 (39)	41 (35)	0.663

TIA, transient ischemic attack; TE, thromboembolism; OAC, oral anticoagulation; VKA, vitamin K antagonist; NOAC, novel oral anticoagulant; ACEi, angiotensin-converting enzyme inhibitor; ARB, angiotensin II receptor blocker.

**Table 2:** Long-term outcomes by blood type.

	All (*n* = 1170)	Type O Blood (*n* = 305 (26%))	Type A Blood (*n* = 413 (35%))	Type B Blood (*n* = 333 (28%))	Type AB Blood (*n* = 119 (10%))	*p*
Mean follow-up (months)	17 ± 13	16 ± 13	17 ± 13	18 ± 13	17 ± 13	0.132
MACE, *n* (%)	52 (4.4)	10 (3.3)	16 (3.9)	16 (4.8)	10 (8.4)	0.096
Ischemic stroke, *n* (%)	42 (3.6)	9 (3.0)	14 (3.4)	12 (3.6)	7 (5.9)	0.529
NCT, *n* (%)	5 (0.4)	0 (0.0)	1 (0.2)	3 (0.9)	1 (0.8)	0.266
All-cause mortality, *n* (%)	5 (0.4)	1 (0.3)	1 (0.2)	1 (0.3)	2 (1.7)	0.178

MACE, major adverse cerebrovascular events; NCT, non-cerebral thromboembolism.

**Table 3:** Multivariable Cox regression analysis of long-term clinical outcomes.

	Model 1		Model 2	
	Adjusted HR (95% CI)	*p*	Adjusted HR (95% CI)	*p*
Type AB vs. non-AB blood	2.01 (1.01–4.00)	0.048	-	-
Type O vs. non-O blood	-	-	0.75 (0.38–1.50)	0.422
CHA_2_DS_2_-VASc score	1.02 (0.92–1.22)	0.450	1.07 (0.92–1.23)	0.386
Anticoagulation duration (months)	0.97 (0.94–0.99)	0.008	0.97 (0.94–0.99)	0.008

HR, hazard ratio; CI, confidence interval.

## Data Availability

Data are not available for the protection of personal health information.
